# Supplementary material for: System-level clustering of testosterone-related biomarkers identifies high-risk aging profiles linked to inflammation and renal function
Source: Commun Med (Lond). 2026 Apr 15;6:220. doi: 10.1038/s43856-026-01556-z (PMC13083999; doi:10.1038/s43856-026-01556-z)
Supplement: Supplementary file 2 — Supplemental Information [file 43856_2026_1556_MOESM2_ESM.pdf]

Supplementary Table S1. Clinical Laboratory Measurement Methods and Devices

Variable	Measurement method	Analyzer / manufacturer
Hemoglobin (Hb)	Sodium lauryl sulfate hemoglobin method	XE-5000 (Sysmex, Japan)
Hematocrit (Hct)	Sheath flow DC method	XE-5000 (Sysmex, Japan)
Albumin (Alb)	Bromocresol purple method	Hitachi 7600 / LABOSPECT 008 (Hitachi, Japan)
Creatinine (Cre)	Enzymatic method	Hitachi 7600 / LABOSPECT 008 (Hitachi, Japan)
Aspartate aminotransferase (AST)	Enzymatic method	Hitachi 7600 / LABOSPECT 008 (Hitachi, Japan)
Alanine aminotransferase (ALT)	Enzymatic method	Hitachi 7600 / LABOSPECT 008 (Hitachi, Japan)
Glucose (Glu)	Enzymatic method	Hitachi 7600 / LABOSPECT 008 (Hitachi, Japan)
Alkaline phosphatase (ALP)	Enzymatic method	Hitachi 7600 / LABOSPECT 008 (Hitachi, Japan)
C-reactive protein (CRP)	Latex agglutination turbidimetric immunoassay	Hitachi 7600 / LABOSPECT 008 (Hitachi, Japan)
Triglycerides (TG)	Enzymatic colorimetric method	Hitachi 7600 / LABOSPECT 008 (Hitachi, Japan)
High-density lipoprotein cholesterol (HDL-C)	Direct method	Hitachi 7600 / LABOSPECT 008 (Hitachi, Japan)
Low-density lipoprotein cholesterol (LDL-C)	Direct method	Hitachi 7600 / LABOSPECT 008 (Hitachi, Japan)
Total testosterone (TT)	Chemiluminescent immunoassay	Architect i2000SR (Abbott, Japan)
Luteinizing hormone (LH)	Electrochemiluminescence immunoassay	Modular E / cobas e 801 (Roche Diagnostics)
Follicle-stimulating hormone (FSH)	Electrochemiluminescence immunoassay	Modular E / cobas e 801 (Roche Diagnostics)

All laboratory measurements were performed using standardized clinical assays routinely applied in Japan.

This table summarizes the analytical methods and instruments used for hematological, biochemical, inflammatory, lipid, and reproductive hormone measurements to ensure reproducibility of the study results.

Supplementary Table S2. Variable-specific missingness after inclusion of subjects with complete age and BMI data (N = 5,854)

Variable	Total N	Missing, n	Missing, %	Non-missing, n
LDL-C	5,854	4,282	73.1	1,572
HDL-C	5,854	3,916	66.9	1,938
TG	5,854	3,419	58.4	2,435
ALP	5,854	2,195	37.5	3,659
Alb	5,854	1,497	25.6	4,357
FSH	5,854	1,363	23.3	4,491
LH	5,854	1,243	21.2	4,611
Glu	5,854	1,160	19.8	4,694
CRP	5,854	1,021	17.4	4,833
ALT	5,854	203	3.5	5,651
AST	5,854	180	3.1	5,674
Cre	5,854	168	2.9	5,686
Hb	5,854	168	2.9	5,686
Hct	5,854	168	2.9	5,686
BW	5,854	0	0.0	5,854
TT	5,854	0	0.0	5,854
Height	5,854	0	0.0	5,854

Variable-specific missingness for clinical and biochemical variables after restricting the analytical cohort to subjects with complete age and body mass index (BMI) data (N = 5,854). Missingness varied substantially across variables, reflecting routine clinical testing practices. Lipid-related and gonadotropin measurements showed higher missing rates, whereas key variables central to the clustering analyses, including total testosterone, inflammatory markers, and renal function indices, exhibited low levels of missingness.

Supplementary Table S3.Correlations between total testosterone quartiles and physiological parameters within each cluster.

cluster	TT_quartile	variable	N	r	p_value
0	Q1	age	489	-0.14878	0.000967
0	Q1	BW	489	0.05151	0.255577
0	Q1	BMI	489	0.049486	0.274759
0	Q1	Hb	471	0.361004	6.05E-16
0	Q1	Hct	471	0.38456	4.76E-18
0	Q1	AST	468	-0.13771	0.002832
0	Q1	ALT	468	-0.08714	0.059598
0	Q1	HDL-C	191	-0.24732	0.000562
0	Q1	LDL-C	147	-0.17583	0.033147
0	Q2	age	709	0.022148	0.556017
0	Q2	BW	709	-0.06209	0.098545
0	Q2	BMI	709	-0.07386	0.049321
0	Q2	Hb	685	0.022735	0.5525
0	Q2	Hct	685	0.011948	0.754933
0	Q2	AST	683	-0.03698	0.334555
0	Q2	ALT	676	-0.07729	0.044558
0	Q2	HDL-C	224	0.184955	0.005492
0	Q2	LDL-C	178	-0.05717	0.448433
0	Q3	age	619	0.032632	0.417678
0	Q3	BW	619	-0.02781	0.489727
0	Q3	BMI	619	-0.01419	0.724535
0	Q3	Hb	608	0.053581	0.187028
0	Q3	Hct	608	0.02401	0.554592
0	Q3	AST	609	0.050409	0.214157
0	Q3	ALT	605	-0.00794	0.845564
0	Q3	HDL-C	229	0.120764	0.068126
0	Q3	LDL-C	189	0.076626	0.294642
0	Q4	age	478	-0.05265	0.250646
0	Q4	BW	478	0.005961	0.896581
0	Q4	BMI	478	0.002967	0.948417

0	Q4	Hb	467	0.066477	0.151479
0	Q4	Hct	467	0.074863	0.10615
0	Q4	AST	461	-0.00622	0.894054
0	Q4	ALT	460	-0.04406	0.345751
0	Q4	HDL-C	176	0.174147	0.020802
0	Q4	LDL-C	140	-0.07056	0.40741
1	Q1	age	259	-0.05054	0.418001
1	Q1	BW	259	0.030037	0.630393
1	Q1	BMI	259	-0.00965	0.877234
1	Q1	Hb	257	0.13337	0.03258
1	Q1	Hct	257	0.138838	0.026035
1	Q1	AST	255	-0.12165	0.052345
1	Q1	ALT	255	-0.09734	0.121027
1	Q1	HDL-C	101	0.084736	0.399505
1	Q1	LDL-C	67	0.006515	0.958273
1	Q2	age	107	0.004563	0.962792
1	Q2	BW	107	-0.03819	0.69612
1	Q2	BMI	107	-0.08165	0.40313
1	Q2	Hb	106	0.130218	0.183369
1	Q2	Hct	106	0.125061	0.201484
1	Q2	AST	106	-0.14203	0.146426
1	Q2	ALT	105	-0.13618	0.16598
1	Q2	HDL-C	40	0.248498	0.122064
1	Q2	LDL-C	26	0.21525	0.290955
1	Q3	age	84	0.115558	0.295217
1	Q3	BW	84	-0.13987	0.204447
1	Q3	BMI	84	-0.11378	0.302739
1	Q3	Hb	84	0.079937	0.469795
1	Q3	Hct	84	0.124527	0.259063
1	Q3	AST	83	0.058084	0.601958
1	Q3	ALT	83	0.065327	0.557369
1	Q3	HDL-C	36	0.273693	0.106264
1	Q3	LDL-C	24	-0.26724	0.206794
1	Q4	age	78	-0.06777	0.555521
1	Q4	BW	78	-0.25284	0.025521

1	Q4	BMI	78	-0.19243	0.091434
1	Q4	Hb	77	-0.04219	0.715628
1	Q4	Hct	77	-0.07379	0.523602
1	Q4	AST	77	0.13805	0.231185
1	Q4	ALT	77	0.111086	0.336145
1	Q4	HDL-C	29	-0.15484	0.42254
1	Q4	LDL-C	23	-0.10246	0.64177
2	Q1	age	1		
2	Q1	BW	1		
2	Q1	BMI	1		
2	Q1	Hb	1		
2	Q1	Hct	1		
2	Q1	AST	1		
2	Q1	ALT	1		
2	Q1	HDL-C	1		
2	Q1	LDL-C	1		
2	Q2	age	0		
2	Q2	BW	0		
2	Q2	BMI	0		
2	Q2	Hb	0		
2	Q2	Hct	0		
2	Q2	AST	0		
2	Q2	ALT	0		
2	Q2	HDL-C	0		
2	Q2	LDL-C	0		
2	Q3	age	0		
2	Q3	BW	0		
2	Q3	BMI	0		
2	Q3	Hb	0		
2	Q3	Hct	0		
2	Q3	AST	0		
2	Q3	ALT	0		
2	Q3	HDL-C	0		
2	Q3	LDL-C	0		
2	Q4	age	0		

2	Q4	BW	0		
2	Q4	BMI	0		
2	Q4	Hb	0		
2	Q4	Hct	0		
2	Q4	AST	0		
2	Q4	ALT	0		
2	Q4	HDL-C	0		
2	Q4	LDL-C	0		
3	Q1	age	719	-0.20753	1.95E-08
3	Q1	BW	719	0.063421	0.089256
3	Q1	BMI	719	0.048386	0.195003
3	Q1	Hb	692	0.298605	1.02E-15
3	Q1	Hct	692	0.30765	1.23E-16
3	Q1	AST	692	-0.04903	0.19767
3	Q1	ALT	689	-0.00851	0.82362
3	Q1	HDL-C	232	-0.09149	0.164849
3	Q1	LDL-C	212	-0.10136	0.141313
3	Q2	age	644	0.037224	0.345609
3	Q2	BW	644	-0.0957	0.015125
3	Q2	BMI	644	-0.07263	0.065489
3	Q2	Hb	624	0.022072	0.582092
3	Q2	Hct	624	0.033518	0.403252
3	Q2	AST	622	-0.03967	0.323236
3	Q2	ALT	618	-0.04634	0.250018
3	Q2	HDL-C	187	0.085338	0.245529
3	Q2	LDL-C	156	0.075828	0.34679
3	Q3	age	763	0.019131	0.597756
3	Q3	BW	763	-0.02556	0.480882
3	Q3	BMI	763	-0.04885	0.17768
3	Q3	Hb	741	-0.03323	0.366339
3	Q3	Hct	741	-0.01351	0.713509
3	Q3	AST	742	0.02535	0.490531
3	Q3	ALT	739	-0.00057	0.98767
3	Q3	HDL-C	202	-0.08253	0.242946
3	Q3	LDL-C	172	0.160064	0.035953

3	Q4	age	904	0.015039	0.651572
3	Q4	BW	904	-0.05838	0.079373
3	Q4	BMI	904	-0.07205	0.030297
3	Q4	Hb	873	0.012581	0.710492
3	Q4	Hct	873	0.015977	0.637335
3	Q4	AST	875	0.035954	0.28807
3	Q4	ALT	875	0.00886	0.793541
3	Q4	HDL-C	290	-0.01073	0.855626
3	Q4	LDL-C	237	0.096112	0.140152

Cluster: K-means clustering–derived subgroup (0–3)

TT quartile: Total testosterone quartile based on cohort-wide distribution

Variable: Physiological or biochemical parameter

N: Number of subjects included in the correlation analysis

Spearman r: Spearman's rank correlation coefficient

P value: Two-sided p value for Spearman correlation

TT quartiles were defined as follows:

Q1: 0.04–2.82 ng/mL

Q2: 2.83–4.42 ng/mL

Q3: 4.43–6.04 ng/mL

Q4: 6.05–30.20 ng/mL

Cluster 2 represents a single-subject exploratory subgroup and is presented for completeness only; no inferential interpretation was applied.

TT, total testosterone; BMI, body mass index; Hb, hemoglobin; Hct, hematocrit;

AST, aspartate aminotransferase; ALT, alanine aminotransferase;

ALP, alkaline phosphatase; Cre, creatinine; CRP, C-reactive protein;

TG, triglycerides; HDL-C, high-density lipoprotein cholesterol;

LDL-C, low-density lipoprotein cholesterol; LH, luteinizing hormone;

FSH, follicle-stimulating hormone.

Expanded_Glossary_Clustering_and_Network_Analysis

Term	Definition
Serum Total Testosterone (TT)	Total testosterone concentration measured in serum, used as the central hormonal variable in multivariate physiological analyses.
Aging-related Physiological Heterogeneity	Inter-individual variation in endocrine, metabolic, inflammatory, and renal parameters associated with aging that is not fully explained by chronological age alone.
System-level Profile	A configuration of multiple physiological markers considered jointly, reflecting coordinated patterns across biological systems rather than isolated abnormalities.
Exploratory Analysis	An analytical approach aimed at describing patterns and heterogeneity without hypothesis testing or causal inference.
Data-driven Approach	An analytical framework that identifies patterns directly from observed data without prespecifying disease categories or hypotheses.
Latent Physiological Pattern	An underlying multivariate structure inferred from biomarker data that is not directly observable from individual variables.
Aging-related Variation	Differences in physiological parameters observed with aging, emphasizing heterogeneity rather than uniform progression.
Hierarchical Clustering	An unsupervised clustering method that iteratively groups individuals based on

	similarity, producing a dendrogram used here for exploratory visualization.
Ward Linkage	A hierarchical clustering criterion that merges clusters by minimizing the increase in within-cluster variance at each step.
K-means Clustering	An unsupervised partitioning algorithm that assigns individuals to one of K clusters by minimizing within-cluster sum of squares in the full standardized feature space.
Elbow Method	A heuristic for selecting the number of clusters by identifying a point where further increases in K yield diminishing reductions in within-cluster sum of squares.
Silhouette Coefficient	A diagnostic metric assessing clustering structure by comparing within-cluster similarity to between-cluster separation, used as a supporting indicator rather than a decision rule.
Column-wise Mean Imputation	A missing data handling method in which missing values are replaced by the mean of the corresponding variable across the cohort.
Adjusted Rand Index (ARI)	A chance-corrected measure of agreement between clustering results, used to assess robustness across imputation strategies.
Principal Component Analysis (PCA)	A dimensionality reduction technique that projects multivariate data onto orthogonal components capturing maximal variance, applied solely for visualization.
Pearson's Correlation Coefficient	A measure of linear association between two continuous variables, used for exploratory assessment of cluster-specific relationships.
Correlation Network	A graphical representation in which variables are depicted as nodes and

	pairwise correlations exceeding a predefined threshold are depicted as edges.
Network Centrality	A descriptive measure of the relative importance of nodes within a network based on their position and connections.
Degree Centrality	The number of direct connections associated with a node, reflecting its relative importance within the network.
Eigenvector Centrality	A measure of node influence that accounts for both the number and importance of its connections.
Betweenness Centrality	A measure indicating how frequently a node lies on the shortest paths between other nodes, reflecting its potential role as a connector.
Force-directed Layout	A network visualization algorithm that positions nodes based on simulated attractive and repulsive forces to support qualitative interpretation.
TT Quartiles (Q1–Q4)	Data-driven stratification of serum total testosterone levels into four groups used for cluster-specific analyses.
External Validation	Evaluation of whether biomarker-defined profiles identified in the primary cohort show consistent associations in an independent dataset.
Cancer Prevalence	The proportion of individuals reporting a history of cancer, used as a system-level clinical outcome for validation.
Biomarker-defined Profile	A classification based on predefined clinical thresholds for TT, CRP, and serum creatinine corresponding to cluster characteristics.
Cluster-specific Profile	A subgroup identified by unsupervised clustering characterized by a distinct

	distribution of endocrine, inflammatory, metabolic, and renal markers.
Singleton Cluster	A cluster consisting of a single individual, retained for completeness but excluded from inferential statistical analyses.
Centrality Patterns	Differences in the relative importance of variables within correlation networks across clusters, reflecting heterogeneity in multivariate structure.
Breakpoint	An estimated point at which the relationship between age and a biomarker changes slope in piecewise linear regression.
LOESS Smoothing	A non-parametric method used to visualize non-linear trends in age-related biomarker trajectories.
Descriptive Configuration	A pattern of biomarker distributions interpreted as a statistical description rather than a disease entity or causal mechanism.
Network-informed Interpretation	An interpretive framework that considers biomarkers as part of interconnected physiological systems rather than independent variables.
Cross-sectional Design	A study design based on measurements at a single time point, limiting inference regarding temporal sequence or causality.
Systems-level Perspective	An approach emphasizing interactions among multiple physiological systems in understanding aging-related variation.
